# Generation WhatsApp: inter-brain synchrony during face-to-face and texting communication

**DOI:** 10.1038/s41598-024-52587-2

**Published:** 2024-02-01

**Authors:** Linoy Schwartz, Jonathan Levy, Olga Hayut, Ofir Netzer, Yaara Endevelt-Shapira, Ruth Feldman

**Affiliations:** 1 Center for Developmental, Social, and Relationship Neuroscience, Reichman University, Herzliya, Israel; 2 Department of Criminology and Gonda Brain Research Center, Bar-Ilan University, Ramat-Gan, Israel; 3 Child Study Center, Yale University, New Haven, USA

**Keywords:** Social behaviour, Social neuroscience

## Abstract

Texting has become one of the most prevalent ways to interact socially, particularly among youth; however, the effects of text messaging on social brain functioning are unknown. Guided by the *biobehavioral synchrony* frame, this pre-registered study utilized hyperscanning EEG to evaluate interbrain synchrony during face-to-face versus texting interactions. Participants included 65 mother-adolescent dyads observed during face-to-face conversation compared to texting from different rooms. Results indicate that both face-to-face and texting communication elicit significant neural synchrony compared to surrogate data, demonstrating for the first time brain-to-brain synchrony during texting. Direct comparison between the two interactions highlighted 8 fronto-temporal interbrain links that were significantly stronger in the face-to-face interaction compared to texting. Our findings suggest that partners co-create a fronto-temporal network of inter-brain connections during live social exchanges. The degree of improvement in the partners' right-frontal-right-frontal connectivity from texting to the live social interaction correlated with greater behavioral synchrony, suggesting that this well-researched neural connection may be specific to face-to-face communication. Our findings suggest that while technology-based communication allows humans to synchronize from afar, face-to-face interactions remain the superior mode of communication for interpersonal connection. We conclude by discussing the potential benefits and drawbacks of the pervasive use of texting, particularly among youth.

## Introduction

Current adolescents mark the first generation to grow up in a technological world. Technologically-assisted communication defines their most natural mode of social connection with peers, is used daily and in abundance, and text messaging is particularly favored by adolescents^1^ who text more than any other age group^2^. Adolescents consider texting their preferred mode of communication with peers and texting surpasses any other mode of communication by a large margin: 88% of adolescents reported texting their friends at least occasionally and 55% on a daily basis. In comparison, face-to-face interactions with peers are reported to decline: in 2010, 33% of adolescents reported having daily face-to-face interactions with friends; by 2015, the numbers dropped to 25%^1,3^, and the COVID-19 and the ensuing isolation further increased the use of technology for social communication. Even prior to the COVID-19 pandemic, approximately half of US adolescents reported being almost constantly online^4^. With the closure of schools during the pandemic the use of technology increased^5^ and 97% of youths reported using social media to interact with friends^6^. These data point to a massive shift in the ways adolescents of this generation create and maintain social bonds.

While text messaging has become a prominent way to keep in touch with family and friends, it is still unclear whether social interactions via technology are sufficient to accommodate the human need for closeness. As a social species, humans require social interactions to sustain their physical and emotional well-being^7^. This need is especially acute for adolescents, whose social brain undergoes massive reorganization at the transition from childhood to adulthood^8^ and are at greater risk to develop psychopathology and social maladjustment^9^. Authors have suggested that the human brain has expanded across primate evolution through social interactions in the natural ecology^10^, and the synchrony of brain and behavior during live social moments is thought to have played an important role in the evolution of human sociality^11^. Naturalistic face-to-face interactions contain features that support neural synchrony, such as gaze, voice, and the free transfer of biological signals associated with physical co-presence, such as chemosignals or touch^12,13^. In contrast, technology now offers social interactions that are stripped of the physical co-presence components, such as conversations via platforms like Zoom or texting that further conceal the partner's facial expression, body cues, chemosignals, and voice. This provides a challenge when partners attempt to interact or create a social bond^14^. Nevertheless, to our knowledge, no study to date has tested how texting as a form of interpersonal communication affects the connection between two brains and whether humans are even able to synchronize their brains and behavior during texting.

Interbrain synchrony, or the temporal coherence of neural dynamics between two or more brains^15–17^, is a mechanism that has been suggested to play a key role in the maturation of the human brain by sustaining affiliative bonds and social groups^11,18–20^. Interbrain synchrony has been found across human close relationships, including parent‒child^13,20,21^, romantic partners^22^, and close friends^23^, and is stronger among partners sharing an affiliative bond compared to strangers^20,23–25^. Interbrain synchrony is a component of the general *biobehavioral synchrony* mechanism, which describes the coordination of biological and behavioral processes between partners during moments of social contact that support the formation of human attachments^17^.

In the current study, we tested the effects of texting in comparison to naturalistic face-to-face communication on interbrain synchrony between attachment partners, and focused on a key developmental transition, the transition to adolescence. Utilizing hyperscanning EEG methodology, we tested mother-adolescent brain-to-brain synchrony during two ecologically valid interactions: face-to-face conversation and texting from different rooms.

Mothers-adolescent dyads were chosen for this study based on previous research that showed strong interbrain synchrony between mothers and their children across ages^13,20,21^. According to the *biobehavioral synchrony* perspective, the mother–child bond is the first context where interbrain and behavioral synchrony are practiced and mastered^12,26,27^. Mothers and children are familiar with each other’s social cues, and for this reason we expected that they may be able to synchronize even via text messages—a medium that lacks voice, gaze, body cues, and other information that transfers during face-to-face interactions—and selected to begin research on interbrain synchrony during texting with mother–child pairs. Texting interactions are common between adolescents and their parents, and data on adolescent-parent texting habits show that over 90% of parents text their children^28^. Texting allows parents to give adolescents greater freedom as they can always be reached by texting^29^, making parent‒child texting interactions a well-practiced form of social communication. For adolescents, texting a parent provides the opportunity not only to update on their whereabouts but also to obtain information and ask for help, and adolescents are reported to prefer texting with their mothers^30^.

We defined our pre-registered regions of interest (ROIs) based on prior two-brain studies. fNIRS studies of mother–child and mother-adolescent neural synchrony reported mainly frontal-frontal homologous synchrony during naturalistic interactions across a range of tasks, such as naturalistic conversation or problem solving^25,31–33^. Temporo-parietal brain-to-brain synchrony during mother–child free conversation or play interactions has also been reported^31,34^. Hyperscanning EEG studies of other affiliative bonds, such as romantic partners or close friends, similarly found temporal-temporal neural synchrony during free interactions^22,23^. Finally, in adolescent-mother conversations, a rich network of fronto-temporal interbrain links has been detected during face-to-face interactions^13^. Overall, this body of work suggests that frontal and temporal areas may play a crucial role in forming interbrain synchrony during social interactions, and therefore these areas were the focus of our study.

Three hypotheses were formulated. First, we hypothesized that both face-to-face and texting interactions would elicit interbrain synchrony relative to a surrogate data condition, as both interactions involve social communication. Second, consistent with prior research^13^, we hypothesized that the face-to-face interaction would facilitate significantly more interbrain connections across the fronto-temporal network compared to the texting interaction. We considered three types of interbrain links: (a) homologous links (same brain region, same hemisphere), (b) cross-brain links (same brain region, different hemisphere), and (c) multidimensional links (cross-region and cross-hemisphere). Consistent with the embeddedness of neural synchrony within moments of behavioral synchrony proposed by the *biobehavioral synchrony* model^17,18^, partners' social behavior was micro-coded at a second-to-second level and focused on episodes of shared positive affect in light of research showing greater positive affect following face-to-face compared to texting interactions^35^. Consistent with prior research that pointed to the connection between neural synchrony and behavioral synchrony^13,20,22,23,36^, we predicted associations between mother-adolescent affect synchrony and the improvement in neural synchrony in the face-to-face relative to the texting interaction.

## Materials and methods

The study has been preregistered—https://osf.io/8kf4c/.

### Participants

The participants were 140 individuals, comprising 70 mother-adolescent pairs, who were recruited through ads posted in community centers and via social media. Of the 70 dyads, 65 dyads had sufficient data to be analyzed in the study. The demographical background was collected using questionnaires, which 61 dyads completed. Based on the self-reporting questionnaires, all the mothers were the biological mothers and primary caregivers of their children, and 86% lived in the same household as the adolescents’ fathers (57 mothers answered). The mothers’ average age was 43.78 years old (SD = 4.48), and the adolescents’ average age was 12.28 years old (SD = 1.22). 45.45% of the children were males (55 answered), and 65%of the children were firstborns (60 answered). All participants were healthy, and all adolescents attended state-controlled typical schools.

The Reichman University institutional ethics committee approved the experiment, and all experiments were performed in accordance with the relevant guidelines and regulations. All mothers signed a written informed consent form for themselves and their adolescent children. All procedures were explained to the participants prior to the experiment, and the participants were free to leave the experiment at any time with full compensation. Participants were reimbursed for study participation ($30 per hour).

### Procedure

The study took place in two adjacent experiment rooms and included two paradigms recorded using dual-EEG. The paradigms were counterbalanced across participants and consisted of positive-valence 3-minute naturalistic interactions. In each paradigm, the mother and adolescent were instructed to engage in a conversation about a positive topic, pre-determined by the researcher. The topic of each conversation was counterbalanced across participants and paradigms, and the possible topics included the planning of a fun day together, a camping trip, or an amusement park (overall three possible topics).

In the face-to-face paradigm, the mother and adolescent set in the same room, 50 cm apart from each other, facing each other, and engaged in a free conversation about the positive topic of request. During the texting paradigm, the participants sat in two adjacent rooms in front of a 24″ S2415H DELL screen with 1920*1080 resolution and 60-frames-per-second refresh rate and communicated by texting using the WhatsAppWeb app, installed on both computers. Using the WhatsAppWeb app, the participants engaged in a texting interaction about the positive topic of request. During the texting interaction, the computers were wired and connected to the same internal network to ensure optimal connection, and a senior technician was present in all experiments to ensure quality control. From each paradigm, the first 2 min were analyzed, consistent with prior research^13,20^.

### Dual neural and behavioral data acquisition

The EEG activity of both the mother and adolescent was recorded simultaneously and continuously throughout the experiment. Data acquisition was performed using a 64-channel BrainAmp amplifier from Brain Products Company (Germany). The EEG system was composed of two Brain product standard subtemporal BrainCap with an integrated chin belt. Each cap included 32 electrodes each, buttoned directly to the cap and arranged according to the international 10/10 system, an extension of the standard 10/20 system (See Supplementary Table S1 for full list of electrodes and electrode positions. Theta/Phi coordinates are reported, standardized to a Theta of 90 for the plane through Fpz, T7, T8, Oz). Analog 0.1–500 Hz band-pass was used for filtering, and data was sampled at 1000 Hz. Impedances were maintained below 10 kOhm, and the ground electrode was placed on the AFz electrode. Both helmets were connected to the same amplifier to ensure millisecond-range synchrony between the EEG recording of the mother and adolescent.

### EEG preprocessing

Preprocessing was conducted using Spyder 5.05 and Python 3.8, utilizing MNE (v0.17.0). First, the EEG data file of each dyad was separated into two data files, one for the mother and one for the adolescent, so that each file could undergo separate preprocessing. Data were then average-referenced, and a 1–50 Hz bandpass filter was applied to all data files, consistent with prior studies^13,23^. Next, the data were segmented into 1000 ms epochs with 500 ms overlap between epochs. Autoreject v0.1^37^, an unsupervised algorithm with Bayesian optimization as the threshold method, was utilized to remove trials containing transient jumps in isolated EEG channels and artifacts affecting groups of channels. Following AR, a sample of the first 10 epochs of each participant was visually inspected pre- and post-AR correction to verify the algorithm's output. While AutoReject specializes in excluding trials containing transient jumps in specific channels, systematic physiological artifacts that may affect multiple sensors, such as eye blinks or muscular movements are not optimally removed by AR algorithms. Therefore, ICA was used to remove artifact components from the data. To that end, MNE’s implementations of fastica and CORRMAP^38^ were used to remove systematic physiological artifacts that affected the data. Independent components (IC) were manually selected for exclusion and served as templates for selecting and excluding similar components in all other participants across the two experimental conditions. Such components included non-physiological components, eye blinks, eye movements, and muscle artifacts to control for muscular movements while speaking (see Supplementary Fig. S1). The removal of muscular movement components was of particular importance, as the experimental conditions differed in the type of muscular movements between speaking and texting.

Overall, following preprocessing and cleaning procedures, an average of 184.61 (SD = 45.15) epochs per dyad remained in the face-to-face condition, while an average of 202.76 (SD = 32.6) epochs per dyad remained in the texting condition. Following preprocessing, dyads that did not share a minimum of 30 common epochs in each condition were excluded from the following connectivity analysis, resulting in the exclusion of 3 dyads.

### Connectivity analysis

Interbrain synchrony was calculated using the weighted phase lag index (wPLI), an interbrain connectivity method that has been used in various previous studies of naturalistic social interactions^13,20,36^.

Interbrain connectivity values were calculated for the beta rhythm (13.5–29.5 Hz) based on previous research reporting that beta frequency has been found to play a crucial role in parent‒child exchanges and attachment processes^13,39–41^. Previous interbrain synchrony studies further reported the beta rhythm to sustain communication between partners sharing affiliative bonds such as mother-adolescents, couples and friends^13,23^, and neural synchrony in the beta rhythm was found during both face-to-face and remote social exchanges^13^. Analytic signals were computed using IIR filtering with a Hamming window to avoid distortion and border effects and the Hilbert transform^42^.

Consistent with prior research, we divided the EEG cap into pre-defined areas of interest based on the research hypotheses^13,23,43^. EEG electrodes were grouped into predefined regions of interest^13,22,23^, resulting in a total of 6 ROIs that were examined in this study. Each ROI consisted of 3 electrodes: right frontal (RF–Fp2, F4, F8), left frontal (LF–Fp1, F3, F7), right central (RC–FC2, CP2, C4), left central (LC–FC1, CP1, C3), right temporal (RT–T8, TP10, P8), and left temporal (LT–T7, TP9, P7), with the frontal and temporal areas, in particular, being the focus of this study. The grouping of channels was used to enhance the reliability of region specification and provide a more meaningful and realistic interpretation of the results^44^. Overall, this resulted in a total of 6 ROIs in each brain, resulting in 36 possible combinations of linkage between the mother's and adolescent's ROIs in the comparison to surrogate data (control) analysis. In the main analysis, we a-priori chose to focus only on the 4 ROIs of the fronto-temporal network, leading to a total of 16 ROI combinations that were evaluated, but conducted a follow-up analysis on all 6 ROI (reported in Table S2, Fig S2). The respective wPLI value of the partners' ROIs was calculated as the mean connectivity value of each of the 3 electrodes in one target ROI with each of the 3 electrodes in the second target ROI, resulting in a total of 9 connectivity values averaged for each interbrain link between 2 ROIs.

Of the 70 dyads participating in the experiment, the data files of 2 dyads were corrupted and discarded, and 3 dyads did not share sufficient common epochs following AutoReject and IC rejection, so connectivity could not be measured, resulting in a total of 65 dyads that were included in the analysis.

### Behavioral coding

Each paradigm was coded offline using our well-validated micro-coding scheme. The micro-coding analyzes the interactions second-by-second in a precise way, building upon a previously validated coding scheme^45,46^ that has shown linkage with the brain basis of attachment in both parent^47^ and child^41^ and has been validated in hyperscanning EEG studies in infancy, adolescence, and adulthood^13,20,22^. Coding was conducted by two trained coders, a main coder who had over 300 h of experience with micro-coding, and a second coder for reliability of the current sample. Both coders were blind to the study hypotheses. The Micro-coding was conducted using a computerized system (Mangold Interact, Mangold International GmbH). In the face-to-face paradigm the interactions were recorded from four different cameras that were placed on four walls of the observational room for maximum coverage. In the texting paradigm, the mother was recorded from one camera and the adolescent from three different cameras, set in their respective rooms. The participants’ affect was manually micro-coded for each participant separately along five codes, consistent with prior research: very negative, negative, neutral, positive, and very positive.

*Affect synchrony* was indexed by episodes when both the adolescent and the mother displayed a positive affect, expressed in smiling or laughing during the face-to-face interactions. Affect synchrony was computed as a conditional probability; adolescent displaying a positive affect given mother displaying a positive affect by the Mandgold Interact software, and focused on the harmonic mean duration of the positive synchronous affect. Behavioral coding was available for 60 of the 65 dyads. Therefore, brain-behavior correlations were computed only for the participants with both brain and behavioral coding data.

### Statistical analysis

#### Comparing neural synchrony during social interactions (face-to-face, texting) vs. surrogate data

First, to control for spurious findings, we conducted a validation analysis of the neural connectivity values in each experimental condition relative to a control condition of surrogate data. Our goal was to evaluate whether each interaction (face-to-face, texting) resulted in increased interbrain connectivity values relative to the surrogate data control, consistent with previous literature on two-brain research^13,25,48,49^.

Surrogate data was created for each interaction separately by computing the data of one member of a dyad (mother) with the data of the other member (adolescent) from a different dyad and calculating the wPLI connectivity values for the surrogate dyad. Computing the surrogate dyad's connectivity values was identical to the computation of the connectivity values of the real dyads. This was done for all possible permutations of mother and other-adolescent in each of the other dyads, resulting in 64 different surrogates for each mother. Overall, a total of 4160 surrogate mother-adolescent combinations were created for the 65 dyads in each condition. Then, the 64 surrogate connectivity values computed for each mother were averaged, leading to a single, average surrogate for each mother.

The wPLI connectivity values of the average surrogate data were then examined relative to the data of the real dyads for each interbrain link. To that end, a repeated-measure analysis of variance (ANOVA) was conducted on the real connectivity values (original mother-adolescent dyads) and the surrogate data (averaged across all possible combinations of mother-other-adolescent pairs), with Condition (real connectivity, surrogate data) and ROI (all 36 possible combinations) as within-subject factors. This analysis was conducted for each experimental interaction separately relative to their respective surrogate controls and covered all 6 ROIs – left and right frontal, central and temporal areas in both the mother and adolescent's brains. The Greenhouse–Geisser correction was used to adjust for lack of sphericity in cases where the sphericity assumption was violated.

As this study is, to the best of our knowledge, the first hyperscanning study to evaluate how people synchronize using text messages relative to face-to-face, we chose not to limit the validation analysis to the pre-hypothesized ROIs (fronto-temporal areas). We therefore conducted this analysis on the brain regions that are often reported in hyperscanning literature and include the frontal, central and parieto-temporal areas^13,20,43,50,51^.

#### Comparing interbrain synchrony between face-to-face and texting interactions

Following the validation analysis, which assessed whether each condition elicited greater interbrain synchrony relative to the control (surrogate data), our following and main analysis focused on a direct comparison of the interbrain connectivity values of the two experimental conditions (face-to-face, texting).

Here, consistent with our second hypothesis, we focused only on fronto-temporal interbrain connections, resulting in 4 areas of interest (RT, LT, RF, LF) for each member of the dyad, leading to 16 possible interbrain links between the mother and adolescent's brains. A repeated-measures ANOVA with Condition (face-to-face, texting) and ROI (16 possible combinations) as within-subject factors was used to compare the interbrain connectivity values between the face-to-face and texting interactions. The Greenhouse–Geisser correction was used to adjust for lack of sphericity in cases where the sphericity assumption was violated.

Following, we used a conservative method and applied a set of nonparametric FDR-corrected Wilcoxon signed-rank tests on all 16 possible mother-adolescent ROI combinations to evaluate which of the 16 possible interbrain links facilitated the interbrain synchrony between the two experimental conditions. All results were FDR-corrected to accommodate the 16 comparisons, and only ROI pairs that reached a *p*-value of 0.05 or smaller following FDR correction are reported in the “Results” section.

#### Brain-behavior correlations

Following, brain-behavior Pearson correlations were used to examine whether social behavior affected neural synchrony in either the face-to-face or texting interactions. Here, we chose to focus on the behavioral aspect of affect synchrony – or how positively synchronous the two individuals appeared during the interactions, as assessed using the second-by-second Micro-coding.

Consistent with previous research linking brain-behavior coupling with homologous connectivity patterns^22,23^ and previous findings reporting that the frontal and temporal areas of mother-adolescent dyads correlated with behavior^13^, we a-priori selected to focus on the homolog right-frontal link that has been found to mediate brain-behavior coupling. Next, we evaluated the mother's right-frontal - adolescent's left-temporal link, another interbrain link that has also been found in previous research to link brain and behavior, as well as to facilitate mother-adolescent neural synchrony when interacting both face-to-face and from afar^13^. The brain-behavior correlations were computed between the face-to-face behavioral affect synchrony values and the increase in interbrain connectivity values between the two experimental conditions (wPLI of face-to-face interaction – wPLI of texting interaction = ΔwPLI) in the two interbrain links of interest.

Participants were excluded from the brain-behavior analysis if their synchronous affect values varied by more than 2.5 SD from the mean synchronous affect across all participants. Notably, only 2 participants were excluded from the brain-behavior analysis due to extreme values.

#### Comparing affective synchrony between face-to-face and texting interactions

Finally, a sample of 18 dyads (27.7% of the overall dyads) was randomly selected to assess whether the two experimental conditions differed in the degree of their affect synchrony. Following Shapiro–Wilk test to examine normal distribution, a standard paired-sample t-test was conducted to evaluate possible differences in affect synchrony between the two conditions.

#### Interbrain synchrony and amount of information analyses and correlations

The next analysis addressed the question of whether the amount of information transferred by each member of the dyad differed between the experimental condition, and whether it affected their ability to form interbrain synchrony. To examine the amount of information exchanged in each condition we used two different measurements – the number of words exchanged by each member of the dyad, and the numbers of times each member of the dyad spoke or texted. Both measurements were taken from a random sample of 17 dyads (26% of the analyzed participants) in both the face-to-face and texting conditions.

First, we conducted a repeated-measures ANOVA with Condition (face-to-face, texting) and Participant (mother, adolescent) as within-subject factors to determine whether there was a difference in the amount of conveyable information in each condition. Next, to assess whether the amount of information exchanged in each condition affected the participants’ interbrain synchrony, we used a set of Pearson correlations between the amount of conveyable information within each condition and the interbrain links that were found to be significant in the face-to-face relative to the texting interaction.

## Results

### Comparing neural synchrony during face-to-face and texting interactions relative to surrogate data

#### Texting interaction

Repeated measures ANOVA with Condition (real texting connectivity, surrogate data of interaction) and ROI (all possible 36 combinations) as within-subject variables revealed a main effect for condition (*F*(1, 64) = 132.81, *p* < 0.001, η^2^p = 0.68), indicating an overall improvement in interbrain synchrony when participants communicated via texting relative to control. No main effect was found for ROI (*F*(35, 2240) = 1.47, *p* = 0.13, η^2^p = 0.02), and no interaction between condition and ROI was found (*F*(35, 2240) = 1.11, *p* = 0.31, η^2^p = 0.02). These findings indicate that interbrain connectivity overall improved during the texting interaction relative to the control surrogate data condition.

#### Face-to-face interaction

**Figure 1:**
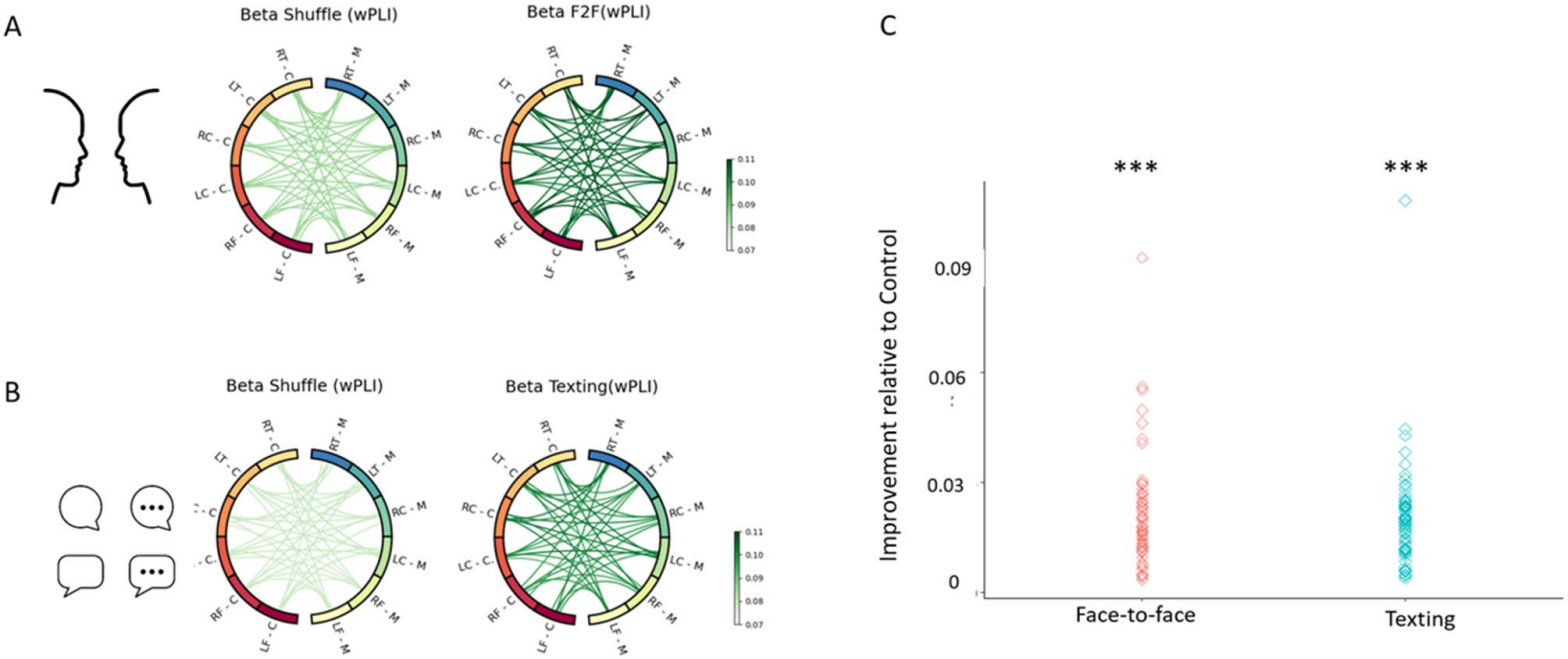
Visualization of validation analysis conducted on face-to-face and texting interactions relative to control (surrogate data): Higher interbrain synchrony was detected during the face-to-face and texting interactions compared to the control condition (surrogate data). (**A**,**B**) Visualization of the surrogate data (left) compared to the real connectivity values (right). Each node represents a different ROI in the brains of the mother and adolescent. *RT* right temporal, *LT* left temporal, *RC* right central, *LC* left central, *RF* right frontal, *LF* left frontal. Darker shades represent greater values of interbrain connectivity (wPLI scores). (**C**) Visualization of the improvement in real interbrain connectivity in each condition relative to control. Each dot represents a dyad. A repeated-measures ANOVA revealed a significant advantage for the face-to-face interaction compared to the control in facilitating interbrain synchrony (*p* < 0.001), and a similar effect was found for the texting interaction condition (*p* < 0.001), showcasing that both social interactions facilitated interbrain connectivity relative to control (surrogate data).

Repeated measures ANOVA with Condition (real face-to-face connectivity, surrogate data of interaction) and ROI (all possible 36 combinations) as within-subject variables revealed a main effect for condition (*F*(1, 64) = 117.78, *p* < 0.001, η^2^p = 0.65), indicating an overall improvement in interbrain synchrony when participants communicated face to face relative to control. No main effect for ROI was found (*F*(35, 2240) = 1.14, *p* = 0.31, η^2^p = 0.02) and no interaction between condition and ROI was found (*F*(35, 2240) = 1.04, *p* = 0.41, η^2^p = 0.02). These results demonstrate increased interbrain connectivity during the face-to-face interaction relative to the control surrogate data condition (see Fig. 1).

After establishing that both face-to-face and texting interactions facilitated greater interbrain synchrony relative to the control (surrogate data) condition, our next analysis focused on the specific interbrain connectivity patterns triggered by each interaction by comparing both conditions directly to each other.

### Comparing neural synchrony between face-to-face and texting interactions

**Figure 2:**
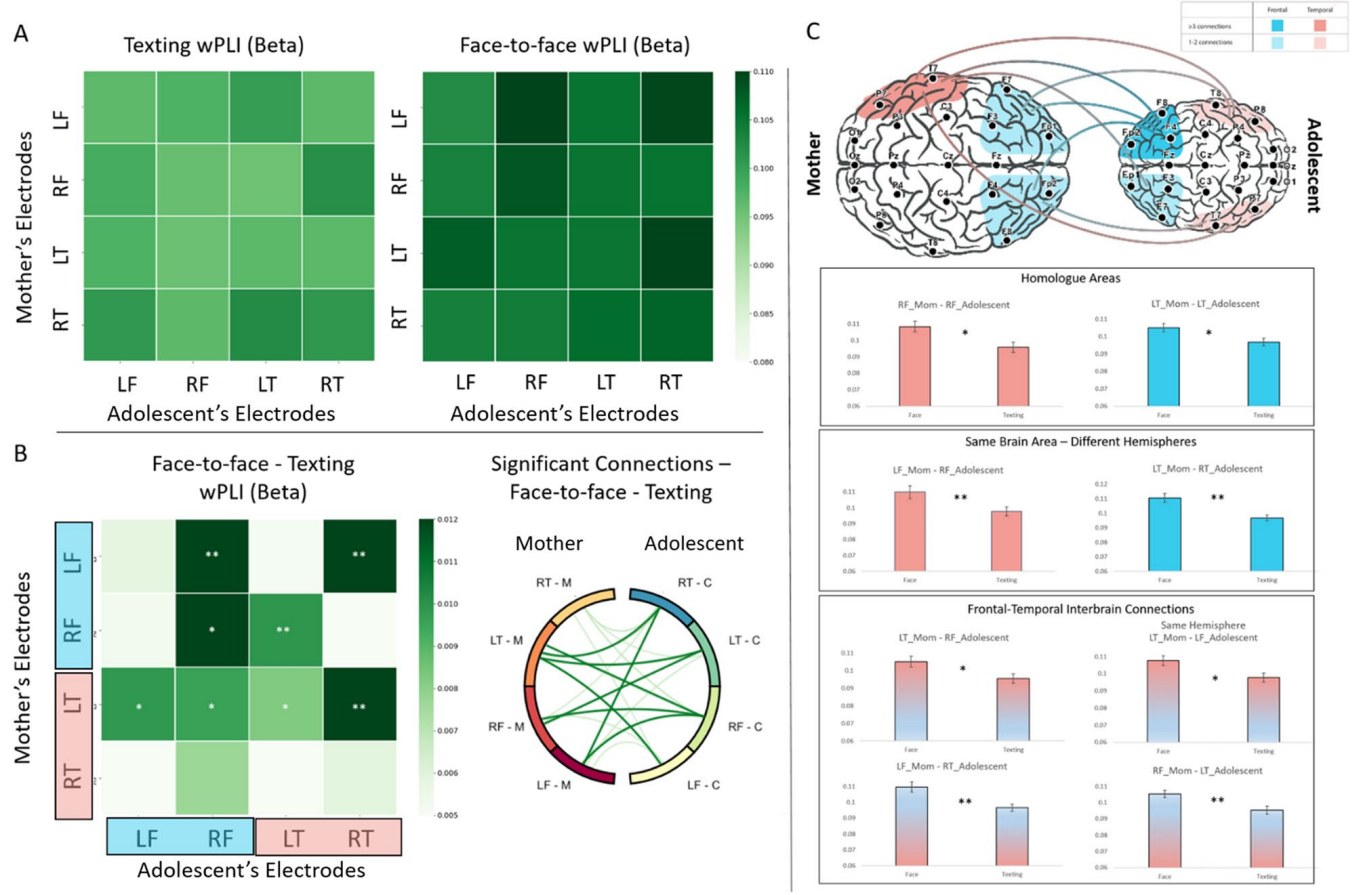
Visualization of significant Interbrain connections between face-to-face and texting interactions: Higher interbrain synchrony was detected during the face-to-face interaction compared to the texting interaction, with 8 interbrain connections emerging when participants interacted face to face, when co-present. *RT* right temporal, *LT* left temporal, *RF* right frontal, *LF* left frontal. (**A**) Visualization of connectivity values (wPLI) during the face-to-face and texting interactions. The x-axis represents the adolescent's brain regions, while the y-axis represents the mother’s brain regions. Darker squares represent links with higher connectivity values. (**B**) Visualization of differences in connectivity values across ROI combinations between face-to-face and texting interactions (left) and the significant interbrain connections that emerged between the two interactions (right). Darker squares represent comparisons with higher connectivity score differences between the face-to-face and texting paradigms. Repeated measures ANOVA revealed a significant main advantage for the face-to-face interaction compared to texting (F(1,64) = 14.35, *p* < 0.001). A set of nonparametric Wilcoxon tests was used to detect differences in wPLI interbrain connectivity measures across each interbrain link. All results were FDR-corrected, and the significant comparisons are marked. On the right, a connectivity circle visualizing the 8 significant interbrain links formed in the face-to-face relative to the texting interaction between the mother and adolescent’s brains. (**C**) All 8 significant interbrain connections found between the mother and adolescent's fronto-temporal network in the face-to-face over texting interaction. The significant comparisons are marked. (*P <0.05 **P <0.01).

Following the validation analysis, we compared the interbrain synchrony between mother-adolescent dyads during the face-to-face and texting interactions using a repeated measures analysis of variance (ANOVA). The test was designed to detect effects stemming from the face-to-face interaction compared to the texting interaction on interbrain connectivity levels. Interbrain synchrony was measured using wPLI scores on interbrain links in the fronto-temporal network of the mother and adolescent. The repeated-measures ANOVA with Condition (face-to-face, texting) and ROI (16 combinations) as within-subject variables revealed a significant main effect for face-to-face compared to texting (*F*(1, 64) = 14.35, *p* < 0.001, η^2^p = 0.18), indicating an overall improvement in interbrain connectivity levels during the face-to-face interaction, when both participants were co-present, relative to the texting interaction. No effect for ROI was found (*F*(15,960) = 0.91, *p* = 0.52, η^2^p = 0.01), and no interaction was found between the condition and ROI (*F*(15,960) = 1.33, *p* = 0.2, η^2^p = 0.02). (See Fig. 2).

Following detection of the main effect of increased interbrain synchrony in the face-to-face over the texting interaction, we next sought to evaluate the pattern of interbrain connectivity between the mother and adolescent's fronto-temporal network. Our following analysis, therefore, focused on detecting the differences in the wPLI connectivity values between the face-to-face and texting conditions in each of the possible 16 links connecting the mother and adolescent's fronto-temporal network. To that end, we used a set of nonparametric Wilcoxon signed-rank tests to evaluate which of the 16 links facilitated the interbrain synchrony pattern observed between the two experimental conditions. All results were FDR-corrected to accommodate 16 comparisons. Significant (*p* < 0.05) interbrain linkages were observed in 8 out of the 16 possible links (Fig. 2, Table 1), in which greater neural synchrony was found during the face-to-face than the texting interactions. These links comprised three subgroups: (a) Homologous linkage between mother and adolescent's frontal and temporal regions, (b) Same-region, cross-hemispheric linkage between mother and adolescent's frontal and temporal regions, and (c) Cross-regional linkage of the mother and adolescent's fronto-temporal brain network.

- *Homologous linkage between the mother's and adolescent's frontal and temporal regions* two homolog links were found: A right-frontal-right-frontal connectivity between the mother and adolescent (T = 637, *z* = 2.85, *p*_(FDR corrected)_ = 0.014), and a left-temporal-left-temporal connectivity between the mother and adolescent (T = 694, *z* = 2.47, *p*_(FDR corrected)_ = 0.031).
- *Same-region, cross-hemispheric linkage between mother and adolescent's frontal and temporal regions.* This included two links: one between the mother's left frontal region and the adolescent's right frontal region (T = 583, *z* = 3.2, *p*_(FDR corrected)_ = 0.007) and the other between the mother's left temporal region and the adolescent's right temporal region (T = 536, *z* = 3.51, *p*_(FDR corrected)_ = 0.004).
- *Cross-region linkage of mother and adolescent's fronto-temporal brain network.* This included four links: first, between the mother's right frontal region and the adolescent's lefttemporal region (T = 598 *z* = 3.1, *p*_(FDR corrected)_ = 0.008). Second, between the mother's left frontal region and the adolescent's right temporal region (T = 515, *z* = 3.64, *p*_(FDR corrected)_ = 0.004). Third, between the mother's left temporal region and the adolescent's right frontal region (T = 725, *z* = 2.27, *p*_(FDR corrected)_ = 0.046), and finally, between the mother's left temporal region and the adolescent's left frontal region (T = 678, z = 2.58, *p*_(FDR corrected)_ = 0.026). (See Fig. 2, Table 1).

**Table 1:** Mean (SD) for face-to-face and texting interactions.

ROI linkage	Face-to-face (wPLI values)	Texting (wPLI values)	*p* (FDR-corrected)
RF_Mother-RF_Adolescent	0.108 (0.03)	0.096 (0.02)	0.014 *
LT_Mother_LT_Adolescent	0.105 (0.02)	0.097 (0.02)	0.031 *
LF_Mother-RF_Adolescent	0.11 (0.03)	0.098 (0.02)	0.007 **
LT_Mother-RT_Adolescent	0.111 (0.02)	0.097 (0.02)	0.004 **
RF_Mother-LT_Adolescent	0.105 (0.02)	0.095 (0.02)	0.008 **
LF_Mother-RT_Adolescent	0.11 (0.03)	0.097 (0.02)	0.004 **
LT_Mother-RF_Adolescent	0.105 (0.03)	0.096 (0.02)	0.046 *
LT_Mother-LF_Adolescent	0.108 (0.02)	0.098 (0.02)	0.026 *

(**C**) All 8 significant interbrain connections found between the mother and adolescent's fronto-temporal network in the face-to-face over texting interaction. The significant comparisons are marked. (*P < 0.05 **P < 0.01).

Following our main pre-registered analysis that focused on the fronto-temporal network, we conducted a post-hoc exploratory analysis, evaluating all 6 possible ROIs. This analysis includes a total of 36 possible interbrain links between the left and right frontal, central and temporal areas of the mother and adolescent’s brains. Overall, the exploratory analysis revealed 11 interbrain links that were found to be significant in the face-to-face relative to the texting condition. The detailed results of the analysis are reported in Supplementary Table S2 and Supplementary Fig. S2. Notably, 7 of the 11 significant links were fronto-temporal connections. Although the number of comparisons in the exploratory analysis increased from 16 to 36, 7 of the 8 significant interbrain links reported in our main fronto-temporal analysis were found even following correction to accommodate 36 comparisons, with the mother’s-left-temporal - adolescent’s-right-frontal interbrain link being the only link that was not found in the follow-up analysis.

### Brain-behavior coupling

#### The increase in interbrain connectivity between face-to-face and texting interactions links with affect synchrony

Consistent with previous literature linking behavior and interbrain connectivity patterns^13,20,22,23^, we assessed brain-behavior links in this study. Here, we examined the correlation between the adolescent-mother synchronous affect (i.e. the average amount of time both mother and adolescent displayed positive affect synchronously during the live interactions) and the increase in interbrain synchrony in the face-to-face over the texting interaction (wPLI of face-to-face interaction – wPLI of texting interaction = ΔwPLI). This was examined on the two significant links of interest: First, the homologous right-frontal link that was found in previous hyperscanning studies of parent‒child dyads to sustain interbrain synchrony^13,25,33^. Second, the mother’s right-frontal - adolescent’s left-temporal link, which has been found in previous research to facilitate mother-adolescent interbrain synchrony when interacting both face-to-face and when communicating remotely^13^. Both links were found to correlate with behavior^13^ and were further found in this study to sustain interbrain synchrony processes when the two interactors were co-present in the face-to-face interaction relative to the texting interaction. Pearson correlation was conducted between the face-to-face behavioral affect synchrony values and the improvement in wPLI connectivity observed between the face-to-face relative to the texting interactions (Δ wPLI) in the two interbrain links of interest.

**Figure 3:**
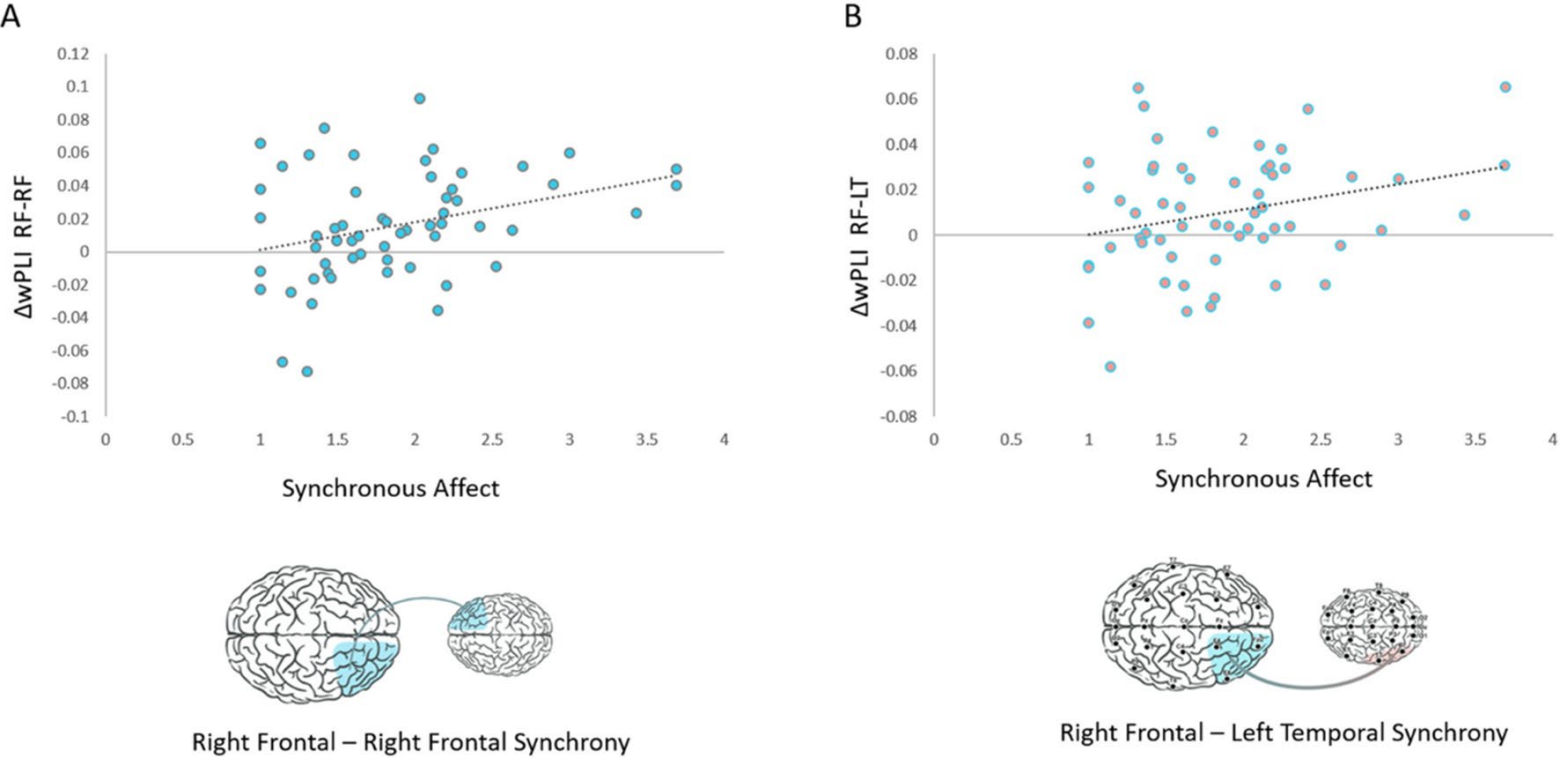
Visualization of correlations between mother-adolescent affect synchrony during the face-to-face interaction and the improvement in interbrain synchrony between face-to-face and texting interactions. (**A**) The contribution of the face-to-face over the texting interaction to interbrain connectivity levels in the homolog right-frontal link (calculated as ΔwPLI, shown on the Y-axis) correlated with the affect synchrony mean duration (X-axis) (*r = 0.27, p = 0.039*). (**B**)  The contribution of the face-to-face over the texting interaction to interbrain connectivity levels in the mother’s right-frontal - adolescent’s left-temporal link (calculated as ∆wPLI, shown on the Y-axis) correlated with the affect synchrony mean duration (X-axis) (*r* = 0.33, *p* = 0.012).

The results revealed that the mean duration of the affect synchrony correlated with the improvement in interbrain synchrony between the face-to-face and the texting interaction in both the homolog right-frontal link (*r* = 0.27, *p* = 0.039), and in the mother's right-frontal - adolescent's left-temporal link (*r* = 0.33, *p* = 0.012). (see Fig. 3).

#### Assessing affect synchrony in face-to-face and texting interactions

Following, we conducted a post-hoc exploratory analysis evaluating the affect synchrony in the texting interaction, as well as the differences in correlations between the raw wPLI values and the synchronous affect in the face-to-face and texting interactions. This analysis was conducted on a sample of 18 dyads (27.7% of the dyads), which were Micro-coded for the texting interaction, in addition to the Micro-coding of the face-to-face interaction.

First, we assessed whether the two experimental conditions (face-to-face, texting) differed in the degree of affect synchrony. As Shapiro–Wilk test indicated the sample distributed normally (p = 0.17), a paired sample t-test was conducted. The results revealed a significantly longer mean synchronous affect in the face-to-face condition (*M* = 1.93, SD = 0.86) over the texting condition (*M* = 0.56, SD = 0.51), (t(17) = 6.53, *p* < 0.001, Cohen's *d* = 1.54).

Next, we assessed the correlations between the raw WPLI values of each interbrain link and the synchronous affect in each condition. Our results revealed that the correlation between raw wPLI values and the synchronous affect were not significant for the RF-RF link in the face-to-face condition (*r'* = 0.26, *p* = 0.3), nor in the texting condition (*r'* =− 0.05, *p* = 0.85). The same pattern of results were observed in the mother's right-frontal - adolescent's left-temporal link, with no correlation found in the face-to-face (*r'* = 0.23, *p* = 0.37), nor in the texting interactions (*r'* =− 0.24, *p* = 0.35). Finally, we assessed the differences between the correlations in the face-to-face and texting interactions in both the homolog right-frontal link and the mother's right-frontal - adolescent's left-temporal link. Following Fisher z transformation, the difference in correlations was not significant in the RF-LT link (Z = 1.28, p = 0.1), nor in the RF-RF link (Z = 0.9, p = 0.19).

### Assessing the link between interbrain synchrony and amount of information exchanged between participants

Finally, to address the question of whether the amount of information exchanged between the participants differed between conditions or affected interbrain synchrony, we conducted a set of follow-up analyses. First, the amount of information exchanged by each participant in each interaction was evaluated using two different measurements – the number of words spoken/written by each participant, and the number of times each participant spoke or texted. Each measurement was then assessed separately in a Repeated-measures ANOVA with Condition (face-to-face, texting) and Participant (mother, adolescent) as within-subject variables. Next, a set of Pearson correlations were used to evaluate whether the amount of information exchanged by the participants in each condition correlated with the 8 interbrain links that were found to be significant in the face-to-face over the texting interaction.

#### An increase in amount of words exchanged in the face-to-face over texting condition

A repeated-measures ANOVA with Condition (face-to-face, texting) and Participant (mother, adolescent) as within-subject variables revealed a main effect for condition (*F*(1, 16) = 240.18, *p* < 0.001, η^2^p = 0.94), indicating that more words were exchanged in the face-to-face than the texting condition. Next, a main effect for participant was found (*F*(1,16) = 22.76, *p* < 0.001, η^2^p = 0.59), indicating that overall the mothers exchanged more words than the adolescents. Finally, no interaction was found between condition and participant (*F*(1, 16) = 2.62, *p* = 0.13, η^2^p = 0.14) (see Supplementary Table S3.1).

#### An increase in the number of times each participant communicate in the face-to-face over texting condition

A repeated-measures ANOVA with Condition (face-to-face, texting) and Participant (mother, adolescent) as within-subject variables revealed a main effect for condition (*F*(1, 16) = 160.09, *p* < 0.001, η^2^p = 0.91), indicating that overall the number of times the participants spoke in the face-to-face condition was greater than the number of times the participants wrote in the texting condition. Next, a main effect for participant was found (*F*(1,16) = 7.54, *p* = 0.014, η^2^p = 0.32), indicating that overall the mothers spoke or wrote more times than the adolescents. Finally, no interaction was found between condition and participant (*F*(1, 16) = 1.77, *p* = 0.2, η^2^p = 0.1) (see Supplementary Table S3.2).

#### No significant correlation between Interbrain synchrony and amount of information exchanged in each condition

The assessment of interbrain synchrony and the amount of information coupling was exploratory and evaluated whether the amount of conveyable information (assessed using two measurements: the number of times each participants communicated, and the number of words exchanged by each participant), correlated with any of the significant interbrain links that were found in the face-to-face over texting interaction. Overall, the analysis resulted in 16 comparisons for each information metric in each condition. Supplementary Table S3.3 reports the correlations between the significant interbrain links and the amount of words exchanged by the mother/adolescent in each condition, and Supplementary Table S3.4 report the correlations between the significant interbrain links and the amount of times the mother/adolescent communicated in each condition. Notably, even before correction to accommodate 16 comparisons per information metric per condition, none of the correlations were significant (*p* > 0.05), suggesting that the conveyable information exchanged in each condition was not found to affect interbrain synchrony in this study.

## Discussion

Texting has become one of the most common modes of social communication by which two or more individuals interact from afar in real-time. Adolescents, in particular, prefer text messaging over any other method of communication, and texting has become their first choice to interact with peers^3^. Despite the growing use of technology-based communication in general and texting in particular, no study to date examined how texting affects the interactors' brains or whether texting could lead to the formation of interbrain synchrony, an important mechanism that underpins the formation of social bonds^11,12,17,18^. To our knowledge, this is the first study to assess whether neural interbrain synchrony could be formed when critical factors such as physical co-presence or visual and auditory aspects of the interaction are omitted.

Several important findings are highlighted by the data. First, we found that the two forms of interpersonal communication examined here, face-to-face and texting, elicited neural synchrony relative to a control condition of surrogate data. While the findings that naturalistic face-to-face interactions trigger interbrain synchrony are not new^13,20,23^, the results for texting demonstrate for the first time that people can form neural synchrony while exchanging text messages. This is despite the fact that texting is a medium of communication deprived of physical presence, voice, and other biobehavioral cues. While our results revealed that texting elicits interbrain connectivity relative to control, the findings also highlight the advantages of face-to-face interactions. Live face-to-face interactions stimulated a rich network of interbrain connections between the partners' fronto-temporal brain regions. Such interbrain links appeared in various formats: homologous same-region-same hemisphere connections, same-region-different-hemisphere connections, and frontal-to-temporal or temporal-to-frontal same and different hemisphere links. Neural synchrony, therefore, increases significantly during social interactions that involve co-presence, consistent with theoretical models suggesting that the human brain is tuned to face-to-face interactions^12,52–54^.

Our findings also show brain-behavior correlations. The degree of affect synchrony between mother and adolescent was associated with the improvement in neural synchrony from texting to face-to-face interactions; the greater the improvement in neural synchrony, the more partners exhibited behavioral synchrony during live interactions. This brain-behavior link was specific to the partners' right-frontal-right-frontal connection, a link found in studies of parent-child interbrain synchrony^13,25,33^. Another neural link associated with brain-behavior correlations was between the mother’s right frontal region and the adolescent’s left temporal region, which was previously found during mother-adolescent face-to-face and remote communication^13^.

Our results add a timely input to the way people in general and adolescents specifically interact socially. Adolescents’ use of text messaging is constantly on the rise: 50% of adolescents reported sending fifty or more texts a day, and one-third reported sending over a hundred messages daily. Reports that consider the difference between texting and face-to-face interactions over the years show that by 2015, more than half of American adolescents reported texting their friends daily, while only one-quarter reported interacting daily with their friends face-to-face^3^. During the COVID-19 pandemic, this trend further escalated, with 97% of adolescents reporting using technology to interact with friends^6^. Text messaging is regarded by adolescents today as a more convenient and easier way of socially communicating^1,55^ despite evidence indicating that face-to-face interactions lead to a more positive affect and greater satisfaction than texting interactions^35^. It appears that the usage of texting as a form of social interaction by teens is high and increasing, underscoring the need to assess the potential benefits and drawbacks of such communication.

We used a dual-EEG hyperscanning method in this study and took a two-brain perspective to evaluate how texting, a relatively novel form of communication, affects behavioral and neural synchrony. Two-brain hyperscanning studies have become a growing trend in neuroscience research over the last decade^15,16,25^, and address different questions than those assessed by traditional social neuroscience experiments that focus on the single brain. Two-brain studies offer a complementary approach to measure in real-time the neural dynamics of two or more brains during ecologically valid naturalistic social exchanges^53,56^.

We focused on the beta rhythm (13.5–29.5 Hz) in light of previous research reporting that beta plays an important role in parent‒child interactions and attachment processes^13,39–41^. Naturalistic interbrain synchrony studies showed that beta synchrony underpins processes of empathy and compassion^57^, sustains communication between attachment partners including couples and friends^23^, and supports mother-adolescent interbrain synchrony during face-to-face and remote interactions^13^. We add to the existing literature that interbrain beta sustains communication even in a medium sparse of information such as texting, where no social cues are available.

Our results open a much-needed discussion on the neural processes that underpin different forms of remote communication. Overall, extensive research has linked the increase in technology use with dire consequences, including depression, anxiety, and poorer mental health^58–62^. Reports further suggested that the desire to connect, coupled with modern technology, may lead to phone addiction that shows similarities to substance addictions^63^. However, alongside these negative outcomes, some studies suggest that in the context of family and friends, texting provides positive aspects. Greater phone use between adolescents and parents is associated with greater family connection^64^. Among friends, online communication may support the traditional benefits of face-to-face interactions, including self-disclosure, validation, companionship, instrumental support, conflict, and conflict resolution^65^, and using text messages as a relationship-maintenance strategy increases closeness^66^. Finally, texting with friends provides emotional relief for distressed adolescents, especially introverts^67^. These results are in line with our findings that interbrain synchrony is evident when communicating using text messages, demonstrating that the great advantages of human synchronization processes could be achieved even when interacting using a sparse medium that includes only text rather than face-to-face interactions.

While our findings show for the first time that synchrony is achieved via texting at the neural level, the data strongly demonstrate that the degree of synchrony during texting is substantially weaker than that occurring during naturalistic interactions that involve physical co-presence and include voice, speech, tone and facial expression. This greater neural connectivity found during face-to-face interactions is evident in the joint adolescent-mother fronto-temporal interbrain network and suggests that social processes may take place in these areas are lacking when partners communicate only via texting. Furthermore, the brain-behavior links that were found between neural synchrony and synchronous affect may suggest a biobehavioral feedback loop, where the increased affect synchrony is linked with increased neural synchrony and vice versa. Therefore, our findings suggest that while texting interactions offer great benefits, such as the opportunity to share feelings and emotions or obtain help or advice from afar in real time, face-to-face interactions remain superior, and we still pay a price when communicating via text messaging instead of face-to-face. Our findings, therefore, contribute to the discussion on the role of texting and the use of technology as a form of interpersonal communication and their impact on adolescents' development and well-being.

Notably, when examining the interbrain connections that emerged during the face-to-face over-texting interactions, from the eight significant fronto-temporal interbrain links, two were homolog connections: the mother-adolescent right-frontal-right-frontal link and mother-adolescent left-temporal-left-temporal link. These homolog connections replicate and expand the results of previous two-brain hyperscanning studies. These studies reported frontal and temporal homolog synchrony emerging between partners during naturalistic interactions, particularly when the partners share an affiliative bond, such as parent‒child, couples, or best friends^13,22,23,25,33,34,49^.

The left temporal area, in which homolog synchrony has been found, is known to be involved in processes of empathy and mentalization, as well as in understanding others' goals and needs^68^. Beta activations in the temporal brain regions have been found in mothers and children and were suggested as neural markers of attachment processes between mother–child dyads^39^. In the context of previous interbrain synchrony studies, temporal neural synchrony has been observed when romantic partners engaged in empathic dialog^23^, when mother–child interacted or played together^34,49^, and when mother-adolescent dyads interacted face-to-face^13^. Our data further show that the mother's left temporal brain region was linked with every single region of the adolescent's brain measured here: the adolescent's right and left frontal and right and left temporal areas (Fig. 2B,C), suggesting that the strong temporal activation found between the mother-adolescent dyads may hint at the underlying attachment processes.

Accordingly, the right-frontal-right-frontal link has been found in multiple fNIRS studies assessing mother-child and mother-adolescent interactions^25,31–33^, as well as in hyperscanning EEG studies that evaluated face-to-face interactions^13^. The frontal cortex is known to be involved in higher-order social functions, including social cognition, mental state knowledge, and social decision-making^69,70^, abilities that are known to develop in the context of maternal care^71^. Here, this link correlated with the mother-adolescent synchronous affect. We suggest that the mother's frontal region may play a role in monitoring the face-to-face interaction and dynamically adjusts to the adolescent's neural processing, processes that may help tune the developing adolescent brain to social life through interbrain mechanisms embedded within coordinated social behavior^11,72^.

Our analysis included two experimental conditions that differ greatly from one another. While texting information communication includes only textual input and few emojis, face-to-face interactions offered rich information that extends beyond the speech content itself, such as facial expressions, tone of voice, body cues, and chemosignals. Our results indicate that face-to-face interactions indeed conveyed more information than texting interactions, in metrics such as the amount of words exchanged and the amount of times each participant communicated. Still, the amount of information did not correlate with interbrain synchrony in any of the significant interbrain links. As such, we suggest that it is unlikely that our results reflect muscular artifacts that were removed by ICA during preprocessing, but rather reflect differences in neural synchrony between the two types of interactions. Such changes could result from multiple factors aspects that differentiate face-to-face co-present interaction and texting.

Evolution led the human species to communicate face-to-face. Technology, being a growing part of our everyday life, is still a new addition, and as the results suggest, may not be sufficient to support the fullness of human interactions. While the evidence demonstrates that texting does allow people to synchronize, face-to-face interactions create a richer network of interbrain connections that are missing when we communicate using text messages.

The brain undergoes significant reorganization during the transition to adolescence that may be particularly notable in areas implicated in social functions, such as the PFC and pSTS^8^. Both areas were found to play a role in the mother-adolescent interbrain synchrony in our study and are part of the fronto-temporal network that underpins socio-cognitive functions^68,73^. We found that during the rapid maturation of these areas in the adolescent brain, moments of naturalistic mother-adolescent interaction triggered not only the two homolog links in the frontal and temporal areas but also a dense net of inter-connections that connected the mother and adolescent's right and left frontal and temporal regions in almost every possible combination. Our results are consistent with previous hyperscanning studies that reported fronto-temporal neural synchrony during social interactions^13,50,74,75^ and further expand the existing literature. The overall increase in connectivity of the fronto-temporal network during face-to-face compared to texting requires much further research to pinpoints the specific maturation of this network over time between two brains in both attached and non-attached social partners.

In summary, text messaging has become a daily practice for nearly everyone nowadays, with billions of users worldwide. This is the first study to assess the brain mechanisms activated during texting. Our findings show interbrain synchrony during texting but also show the co-creation of a rich network of fronto-temporal interbrain connections during face-to-face exchanges that is missing while texting. While texting is a useful method to communicate in real-time and receive support and sympathy from family and friends, there may be a danger in over-reliance on texting, particularly for the developing brain.

### Supplementary Information

Supplementary Information.

## Data Availability

The data generated during the current study are not publicly available due to participants' privacy but are available from the corresponding author on reasonable request.
