# Supplementary material for: Generation WhatsApp: inter-brain synchrony during face-to-face and texting communication
Source: Sci Rep. 2024 Feb 1;14:2672. doi: 10.1038/s41598-024-52587-2 (PMC10834538; doi:10.1038/s41598-024-52587-2)
Supplement: Supplementary file 1 — Supplementary Information. [file 41598_2024_52587_MOESM1_ESM.docx]

**Table S1 – Full list of electrodes and electrodes locations –**

| **Phi** | **Theta** | **Electrode** |
| --- | --- | --- |
| -72 | -90 | Fp1 |
| 72 | 90 | Fp2 |
| -51 | -60 | F3 |
| 51 | 60 | F4 |
| 0 | -45 | C3 |
| 0 | 45 | C4 |
| 51 | -60 | P3 |
| -51 | 60 | P4 |
| 72 | -90 | O1 |
| -72 | 90 | O2 |
| -36 | -90 | F7 |
| 36 | 90 | F8 |
| 0 | -90 | T7 |
| 0 | 90 | T8 |
| 36 | -90 | P7 |
| -36 | 90 | P8 |
| 90 | 45 | Fz |
| 0 | 0 | Cz |
| -90 | 45 | Pz |
| -46 | -31 | FC1 |
| 46 | 31 | FC2 |
| 46 | -31 | CP1 |
| -46 | 31 | CP2 |
| -21 | -69 | FC5 |
| 21 | 69 | FC6 |
| 21 | -69 | CP5 |
| -21 | 69 | CP6 |
| -18 | -113 | FT9 |
| 18 | 113 | FT10 |
| 18 | -113 | TP9 |
| -18 | 113 | TP10 |
| 90 | 67 | Afz (Gnd) |
| 90 | 23 | FCz (original Ref) |

**Table. S1 – Electrodes and electrode placement.** The EEG caps used were Brain product standard subtemporal Caps with integrated chin belt, buttoned directly to the cap. All electrodes were placed according to the international 10-10 system, and their Theta/Phi coordinates are reported, standardized to a Theta of 90 for the plane through Fpz, T7, T8, Oz.

**Figure S1 – Example of components excluded in ICA**

**
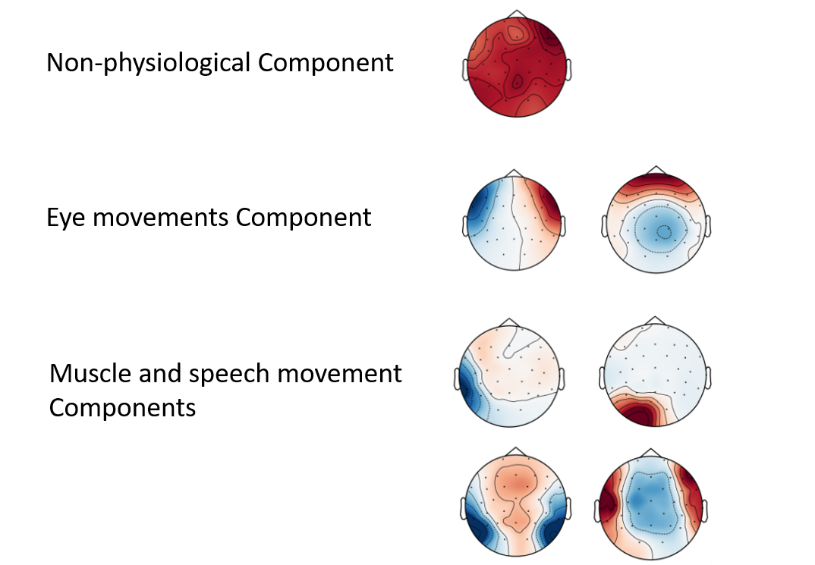
**

**Fig. S1 - Example of components excluded in IC analysis.** MNE's implementations of fastica and CORRMAP have been used to manually inspect and identify different types of components for exclusion. Such independent components (IC) included muscular movement and speech components, eye movement components, and non – physiological components. The identification of the IC components was based on topographies and power spectrum. Following manual inspection, the chosen component for exclusion were used as templates for selecting similar components across all participants and experimental conditions.

**Table S2. Mean (SD) for Face-to-face and Texting Interactions - all 6 ROIs Analysis**

| p (FDR-corrected) | Texting | Face-to-face (wPLI) | ROI linkage |
| --- | --- | --- | --- |
| 0.028 | 0.096 (0.02) | 0.108 (0.03) | RF_Mother-RF_Adolescent |
| 0.048 | 0.097 (0.02) | 0.105 (0.02) | LT_Mother_LT_Adolescent |
| 0.017 | 0.098 (0.02) | 0.11 (0.03) | LF_Mother-RF_Adolescent |
| 0.008 | 0.097 (0.02) | 0.111 (0.02) | LT_Mother-RT_Adolescent |
| 0.017 | 0.095 (0.02) | 0.105 (0.02) | RF_Mother-LT_Adolescent |
| 0.008 | 0.097 (0.02) | 0.11 (0.03) | LF_Mother-RT_Adolescent |
| 0.04 | 0.098(0.02) | 0.108 (0.02) | LT_Mother-LF_Adolescent |
| 0.048 | 0.093 (0.02) | 0.102 (0.03) | LF_Mother-LC_Adolescent |
| 0.028 | 0.098 (0.02) | 0.107 (0.02) | RC_Mother-RF_Adolescent |
| 0.04 | 0.098(0.02) | 0.108 (0.03) | LC_Mother-RF_Adolescent |
| 0.031 | 0.098 (0.02) | 0.108 (0.02) | LT_Mother-LC_Adolescent |

**Table S2 – Analysis of interbrain connectivity in left and right Frontal, Central and Temporal ROIs –** following the pre-hypothesized analysis focusing on the fronto-temporal network of the mother and adolescent’s brains (4 ROIs), we conducted a follow-up exploratory analysis evaluating the interbrain connectivity in all the 6 ROIs, including the central brain area. This resulted in 36 possible links (6x6) instead of the 16 assessed in the main analysis. RT – right temporal, LT – left temporal, RC – right central, LC- left central, RF – right frontal, LF- left frontal. A set of nonparametric Wilcoxon tests was used to detect differences in wPLI interbrain connectivity measures across each interbrain link. All results were FDR-corrected, and the significant comparisons are reported. Of the 8 significant interbrain links that were found in the main analysis, 7 links were significant following FDR correction accommodating 36 instead of 16 comparisons. The single non-significant link that was found in the main analysis was LT-Mother-RF-Adolescent. This analysis further revealed 4 new additional interbrain links were found between central brain regions and frontal or temporal areas: LF-Mother-LC-Adolescent, RC-Mother-RF-Adolescent, LC-Mother-RF-Adolescent and LT-Mother-LC-Adolescent.

**Figure S2 – Visualization of Face-to-face and Texting Interactions - all 6 ROIs Analysis**


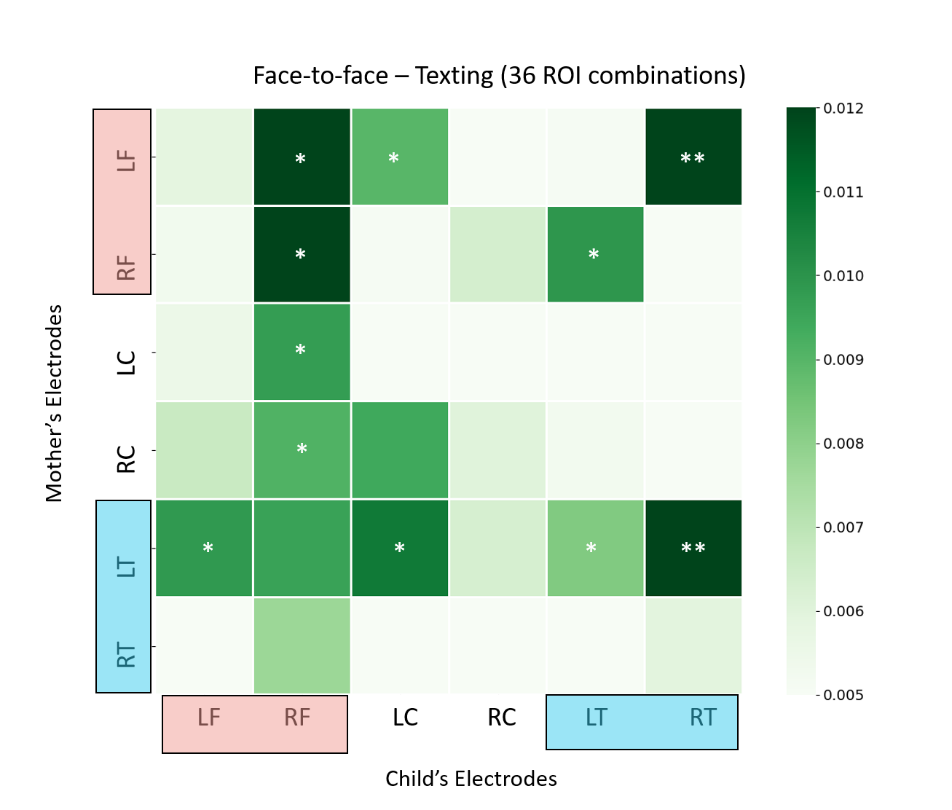


**Fig. S2 - Visualization of significant Interbrain connections between Face-to-face and Texting Interaction (6 ROIs)**: Visualization of differences in connectivity values across ROI combinations between face-to-face and texting interactions. Darker squares represent comparisons with higher connectivity score differences between the face-to-face and texting paradigms. Higher interbrain synchrony was detected during the face-to-face interaction compared to the texting interaction, with 11 interbrain connections emerging when participants interacted face to face, when co-present, than texting. All results were FDR-corrected, and the significant comparisons are marked. (*P <0.05 **P <0.01)

**Table S3 - Amount of information exchanged in each paradigm and correlations between the amount of information exchanged and connectivity across ROIs and conditions**

**S3.1 - Number of words exchanged in each paradigm –**

|  | | | | | | | | | |
| --- | --- | --- | --- | --- | --- | --- | --- | --- | --- |
| **Condition** | | **Participant** | | **Mean** | | **SD** | | **N** | |
| Face-to-Face |  | Child |  | 101.118 |  | 34.097 |  | 17 |  |
|  |  | Mother |  | 147.353 |  | 35.586 |  | 17 |  |
| Texting |  | Child |  | 33.235 |  | 15.213 |  | 17 |  |
|  |  | Mother |  | 57.471 |  | 18.745 |  | 17 |  |
|  | | | | | | | | | |

**S3.2 - Number of times each participant spoke**

| **Condition** | | **Participant** | | **Mean** | | **SD** | | **N** | |
| --- | --- | --- | --- | --- | --- | --- | --- | --- | --- |
| Face-to-Face |  | Child |  | 21.529 |  | 5.735 |  | 17 |  |
|  |  | Mother |  | 22.882 |  | 5.243 |  | 17 |  |
| Texting |  | Child |  | 7.765 |  | 3.212 |  | 17 |  |
|  |  | Mother |  | 8.294 |  | 2.845 |  | 17 |  |

**S3.3 - Correlations between the amount of words spoken by the mother/adolescent and wPLI Connectivity in each experimental condition**

| **Face-to-Face Condition** | | | | | | | |
| --- | --- | --- | --- | --- | --- | --- | --- |
| ***p* (uncorrected)** | **Correlation with amount of words  - Adolescent (r')** | | ***p* (uncorrected)** | | **Correlation with amount of words  - Mother (*r'*)** | **ROI links** | |
| 0.17 | -0.35 | | 0.13 | | 0.385 | LT_mother-RT_child | |
| 0.09 | -0.422 | | 0.4 | | 0.218 | LT_mother-LT_child | |
| 0.2 | -0.324 | | 0.56 | | -0.151 | LT_mother-RF_child | |
| 0.6 | -0.136 | | 0.5 | | -0.177 | LT_mother-LF_child | |
| 0.23 | -0.311 | | 0.57 | | 0.15 | RF_mother-LT_child | |
| 0.11 | -0.401 | | 0.25 | | 0.295 | RF_mother-RF_child | |
| 0.22 | -0.313 | | 0.18 | | 0.343 | LF_mother-RT_child | |
| 0.21 | -0.321 | | 0.43 | | 0.206 | LF_mother-RF_child | |
| **Texting Condition** | | | | | | | |
| ***p* (uncorrected)** | **Correlation with amount of words  - Adolescent (r')** | ***p* (uncorrected)** | | **Correlation with amount of words  - Mother (*r'*)** | | | **ROI links** |
| 0.24 | -0.303 | 0.15 | | -0.362 | | | LT_mother-RT_child |
| 0.54 | 0.159 | 0.49 | | -0.179 | | | LT_mother-LT_child |
| 0.71 | 0.098 | 0.44 | | -0.203 | | | LT_mother-RF_child |
| 0.36 | 0.239 | 0.17 | | -0.347 | | | LT_mother-LF_child |
| 0.85 | -0.051 | 0.79 | | 0.071 | | | RF_mother-LT_child |
| 0.9 | 0.032 | 0.34 | | -0.247 | | | RF_mother-RF_child |
| 0.41 | -0.213 | 0.82 | | 0.06 | | | LF_mother-RT_child |
| 0.39 | 0.223 | 0.62 | | -0.13 | | | LF_mother-RF_child |

**S3.4 - Correlations between the amount of times the mother/adolescent communicated in each condition and wPLI Connectivity**

| **Face-to-Face Condition** | | | | | | | |
| --- | --- | --- | --- | --- | --- | --- | --- |
| ***p* (uncorrected)** | **Correlation with episodes of speak  - Adolescent (r')** | ***p* (uncorrected)** | | | **Correlation with episodes of speak  - Mother (*r'*)** | **ROI links** | |
| 0.26 | -0.29 | 0.94 | | | -0.02 | LT_mother-RT_child | |
| 0.67 | 0.112 | 0.13 | | | 0.383 | LT_mother-LT_child | |
| 0.31 | 0.261 | 0.31 | | | 0.261 | LT_mother-RF_child | |
| 0.99 | -0.002 | 0.9 | | | -0.035 | LT_mother-LF_child | |
| 0.43 | 0.204 | 0.19 | | | 0.334 | RF_mother-LT_child | |
| 0.96 | -0.013 | 0.66 | | | 0.114 | RF_mother-RF_child | |
| 0.5 | 0.177 | 0.13 | | | 0.383 | LF_mother-RT_child | |
| 0.73 | 0.091 | 0.54 | | | 0.16 | LF_mother-RF_child | |
| **Texting Condition** | | | | | | | |
| ***p* (uncorrected)** | **Correlation with episodes of messaging  - Adolescent (r')** | | ***p* (uncorrected)** | **Correlation with episodes of messaging  - Mother (*r'*)** | | | **ROI links** |
| 0.16 | -0.356 | | 0.08 | -0.435 | | | LT_mother-RT_child |
| 0.89 | -0.035 | | 0.62 | -0.128 | | | LT_mother-LT_child |
| 0.67 | -0.111 | | 0.43 | -0.206 | | | LT_mother-RF_child |
| 0.44 | 0.2 | | 0.71 | 0.096 | | | LT_mother-LF_child |
| 0.25 | -0.294 | | 0.34 | -0.249 | | | RF_mother-LT_child |
| 0.86 | -0.046 | | 0.79 | -0.072 | | | RF_mother-RF_child |
| 0.96 | 0.015 | | 0.84 | -0.054 | | | LF_mother-RT_child |
| 0.15 | 0.366 | | 0.32 | 0.255 | | | LF_mother-RF_child |

**Table S3. Amount of information exchanged in each paradigm and interbrain connectivity correlations for each ROI in each Paradigms**

**Tables 3.1 & 3.2** To examine whether the number of words differed between conditions, we assessed the amount of words exchanged by each participant (Table 3.1) and the amount of times each participants spoke or wrote in each interaction. The analyses were conducted on a random sample of 17 dyads from our sample of 65 dyads (over 25%). The results indicate that the amount of information exchanged in each condition differed between the face-to-face and texting conditions, with more words exchanged in the face-to-face condition ((F(1, 16) = 240.18, p < 0.001, η2p = 0.94), and more times each member of the dyad communicated in the face-to-face condition ((F(1, 16) = 160.09, p < 0.001, η2p = 0.91). Notably, an effect was also found for participants in the dyad, with the mother exchanging more words and communicating more times than the adolescent (F(1,16) = 22.76, p < 0.001, η2p = 0.59 and F(1,16) = 7.54, p = 0.014, η2p = 0.32, respectively).

**Tables 3.3 & 3.4** To assess whether the amount of information exchanged in each condition affected IBS, we evaluated the correlations between the interbrain links that were found to be significantly stronger in the face-to-face than texting condition and between two separate parameters evaluating the amount of information exchanged. The parameters were the number of words spoken (in the face-to-face condition) or written (in the texting condition), and the number of times the participants spoke or started texting. Our results indicate that in both conditions the correlations were non-significant (*p* > 0.05) even before correcting to accommodate 16 comparisons per parameter per condition.

**Table S4 – Correlations between Power and Connectivity across ROIs and Paradigms**

**Correlations between Power and Connectivity – Face to Face Paradigm – Mother's ROIs**

| **wPLI Connectivity** | **p value (FDR-corrected)** | **t-value** | **r'** | **Interbrain link** | | **Power** | **ROI** |
| --- | --- | --- | --- | --- | --- | --- | --- |
| 0.106 | 0.094 | -2.321 | -0.281 | RT(Mother)+RT(Adolescent) | | -124.914 | RT(Mother) |
| 0.104 | 0.098 | -2.580 | -0.309 | RT(Mother)+LT(Adolescent) | |  |  |
| 0.104 | 0.189 | -1.924 | -0.236 | RT(Mother)+RF(Adolescent) | |  |  |
| 0.111 | 0.946 | -0.068 | -0.009 | RT(Mother)+LF(Adolescent) | |  |  |
| 0.111 | 0.537 | -0.905 | -0.113 | LT(Mother)+RT(Adolescent) | | -124.316 | LT(Mother) |
| 0.105 | 0.477 | -1.117 | -0.139 | | LT(Mother)+LT(Adolescent) |  |  |
| 0.105 | 0.186 | -2.600 | -0.311 | LT(Mother)+RF(Adolescent) | |  |  |
| 0.108 | 0.321 | -1.492 | -0.185 | LT(Mother)+LF(Adolescent) | |  |  |
| 0.105 | 0.907 | -0.262 | -0.033 | RF(Mother)+RT(Adolescent) | | -126.469 | RF(Mother) |
| 0.105 | 0.066 | -2.576 | -0.309 | RF(Mother)+LT(Adolescent) | |  |  |
| 0.108 | 0.518 | -0.995 | -0.124 | RF(Mother)+RF(Adolescent) | |  |  |
| 0.104 | 0.998 | -0.081 | -0.010 | RF(Mother)+LF(Adolescent) | |  |  |
| 0.110 | 0.427 | -1.257 | -0.156 | LF(Mother)+RT(Adolescent) | | -126.256 | LF(Mother) |
| 0.105 | 0.843 | -0.407 | -0.051 | LF(Mother)+LT(Adolescent) | |  |  |
| 0.110 | 0.320 | -1.576 | -0.195 | LF(Mother)+RF(Adolescent) | |  |  |
| 0.102 | 0.813 | -0.513 | -0.065 | LF(Mother)+LF(Adolescent) | |  |  |

**Correlations between Power and Connectivity – Face to Face paradigm – Adolescent's ROIs**

| **wPLI**  **Connectivity** | **p value (FDR-corrected)** | **t-value** | **r'** | **Interbrain link** | **Power** | **ROI** |
| --- | --- | --- | --- | --- | --- | --- |
| 0.107 | 1.000 | -0.064 | -0.008 | RT(Adolescent)+RT(Mother) | -123.387 | RT(Adolescent) |
| 0.106 | 1.000 | 0.651 | 0.082 | RT(Adolescent)+LT(Mother) |  |  |
| 0.104 | 1.000 | 0.146 | 0.018 | RT(Adolescent)+RF(Mother) |  |  |
| 0.104 | 1.000 | -0.773 | -0.097 | RT(Adolescent)+LF(Mother) |  |  |
| 0.111 | 0.860 | -0.998 | -0.125 | LT(Adolescent)+RT(Mother) | -123.826 | LT(Adolescent) |
| 0.105 | 1.000 | -1.161 | -0.145 | LT(Adolescent)+LT(Mother) |  |  |
| 0.105 | 1.000 | -0.150 | -0.019 | LT(Adolescent)+RF(Mother) |  |  |
| 0.108 | 1.000 | 0.070 | 0.009 | LT(Adolescent)+LF(Mother) |  |  |
| 0.105 | 1.000 | 1.135 | 0.142 | RF(Adolescent)+RT(Mother) | -124.363 | RF(Adolescent) |
| 0.105 | 0.961 | 0.049 | 0.006 | RF(Adolescent)+LT(Mother) |  |  |
| 0.108 | 1.000 | 0.294 | 0.037 | RF(Adolescent)+RF(Mother) |  |  |
| 0.104 | 1.000 | 0.673 | 0.084 | RF(Adolescent)+LF(Mother) |  |  |
| 0.110 | 1.000 | -0.144 | -0.018 | LF(Adolescent)+RT(Mother) | -124.970 | LF(Adolescent) |
| 0.105 | 1.000 | -1.458 | -0.181 | LF(Adolescent)+LT(Mother) |  |  |
| 0.110 | 1.000 | -1.384 | -0.172 | LF(Adolescent)+RF(Mother) |  |  |
| 0.102 | 0.913 | -1.077 | -0.135 | LF(Adolescent)+LF(Mother) |  |  |

**Correlations between Power and Connectivity – Texting Paradigm – Mother's ROIs**

| **wPLI**  **Connectivity** | **p value (FDR-corrected)** | **t-value** | **r'** | | **Interbrain link** | **Power** | **ROI** |
| --- | --- | --- | --- | --- | --- | --- | --- |
| 0.102 | 0.741 | 0.902 | 0.113 | RT(Mother)+RT(Adolescent) | | -126.848 | RT(Mother) |
| 0.097 | 1.000 | 1.139 | 0.142 | RT(Mother)+LT (Adolescent) | |  |  |
| 0.101 | 1.000 | 0.069 | 0.009 | RT(Mother)+RF (Adolescent) | |  |  |
| 0.097 | 0.863 | -0.386 | -0.049 | RT(Mother)+LF (Adolescent) | |  |  |
| 0.097 | 0.737 | 1.502 | 0.186 | LT(Mother)+RT(Adolescent) | | -126.455 | LT(Mother) |
| 0.097 | 1.000 | 0.999 | 0.125 | LT(Mother)+LT (Adolescent) | |  |  |
| 0.096 | 0.897 | 0.969 | 0.121 | LT(Mother)+RF (Adolescent) | |  |  |
| 0.098 | 0.868 | 0.455 | 0.057 | LT(Mother)+LF (Adolescent) | |  |  |
| 0.102 | 0.524 | 1.875 | 0.230 | RF(Mother)+RT(Adolescent) | | -128.654 | RF(Mother) |
| 0.095 | 0.877 | 0.604 | 0.076 | RF(Mother)+LT (Adolescent) | |  |  |
| 0.096 | 0.987 | 0.016 | 0.002 | RF(Mother)+RF (Adolescent) | |  |  |
| 0.098 | 0.932 | 0.469 | 0.059 | RF(Mother)+LF(Adolescent) | |  |  |
| 0.097 | 0.908 | 1.941 | 0.238 | LF(Mother)+RT(Adolescent) | | -128.651 | LF(Mother) |
| 0.100 | 0.942 | -0.223 | -0.028 | LF(Mother)+LT (Adolescent) | |  |  |
| 0.098 | 0.685 | 0.875 | 0.110 | LF(Mother)+RF (Adolescent) | |  |  |
| 0.097 | 0.830 | -0.916 | -0.115 | LF(Mother)+LF (Adolescent) | |  |  |

**Correlations between Power and Connectivity – Texting Paradigm – Adolescent's ROIs**

| **wPLI**  **Connectivity** | **p value (FDR-corrected)** | **t-value** | **r'** | **Interbrain link** | **Power** | **ROI** |
| --- | --- | --- | --- | --- | --- | --- |
| 0.101 | 0.749 | -0.810 | -0.102 | RT(Adolescent)+RT(Adolescent) | -124.760 | RT(Adolescent) |
| 0.102 | 0.709 | -0.698 | -0.088 | RT(Adolescent)+LT (Adolescent) |  |  |
| 0.097 | 0.733 | -1.101 | -0.137 | RT(Adolescent)+RF (Adolescent) |  |  |
| 0.101 | 0.959 | -0.052 | -0.007 | RT(Adolescent)+LF (Adolescent) |  |  |
| 0.097 | 0.783 | -0.545 | -0.069 | LT(Adolescent)+RT(Adolescent) | -124.686 | LT(Adolescent) |
| 0.097 | 0.828 | 1.140 | 0.142 | LT(Adolescent)+LT (Adolescent) |  |  |
| 0.096 | 0.822 | 0.431 | 0.054 | LT(Adolescent)+RF (Adolescent) |  |  |
| 0.098 | 0.814 | -0.835 | -0.105 | LT(Adolescent)+LF (Adolescent) |  |  |
| 0.102 | 1.000 | -1.628 | -0.201 | RF(Adolescent)+RT(Adolescent) | -125.980 | RF(Adolescent) |
| 0.095 | 0.847 | -0.902 | -0.113 | RF(Adolescent)+LT (Adolescent) |  |  |
| 0.096 | 0.775 | -1.314 | -0.163 | RF(Adolescent)+RF (Adolescent) |  |  |
| 0.098 | 1.000 | -1.520 | -0.188 | RF(Adolescent)+LF(Adolescent) |  |  |
| 0.097 | 0.855 | 1.421 | 0.176 | LF(Adolescent)+RT(Adolescent) | -126.233 | LF(Adolescent) |
| 0.100 | 0.949 | 0.215 | 0.027 | LF(Adolescent)+LT (Adolescent) |  |  |
| 0.098 | 0.967 | -0.118 | -0.015 | LF(Adolescent)+RF (Adolescent) |  |  |
| 0.097 | 0.780 | -0.699 | -0.088 | LF(Adolescent)+LF (Adolescent) |  |  |

**Table S4 - Power and Interbrain connectivity correlations for each ROI in each Paradigms were not significant, indicating that power did not affect interbrain Connectivity values.** To validate that the observed differences in wPLI interbrain connectivity are not related to changes in power, beta power spectral density (PSD) was calculated for each ROI of the Mother and Adolescent and correlated with the interbrain connectivity of the relevant ROI. PSD was calculated using MNE's implementation of PSD using multitaper, and PSD scores were calculated for each electrode in each paradigm separately. Next, the power of each ROI was calculated as the average of the 3 relevant electrodes comprising each ROI. The power values of each ROI in each condition (face to face and texting interactions) did not correlate with each of the wPLI connectivity values observed for the relevant ROI. Following correcting to multiple compressions (a total of 16 in each condition. Overall a total of 64 comparisons are reported here), none yielded a significant link between power and interbrain connectivity. Mean correlation between IBS and power in each condition is reported here – Adolescent (texting) – r' = -0.055, Mother (texting) – r' = 0.08, Adolescent (face-to-face) – r' = -0.03, Mother (face-to-face) – r' = -0.16. In conclusion, the analysis revealed that power had no effect on the interbrain connectivity values reported in our study.
